# Children and Multidrug-Resistant Tuberculosis in Mumbai (Bombay), India

**DOI:** 10.3201/eid0811.020513

**Published:** 2002-11

**Authors:** Sunil Karande, Sandeep B. Bavdekar

**Affiliations:** * Lokmanya Tilak Municipal Medical College, Mumbai, India,; † Lokmanya Tilak Municipal General Hospital, Mumbai, India; ‡ Seth Gordhandas Sunderdas Medical College, Mumbai, India; § King Edward VII Memorial Hospital, Mumbai, India

**Keywords:** medication errors, AIDS, tuberculosis, children, India, multidrug-resistant tuberculosis

**To the Editor:** India has the highest number of tuberculosis (TB) cases in the world. Each year in India, over 2 million new cases of TB are diagnosed, and approximately 500,000 persons die of the disease (1). During the last decade, multidrug-resistant TB has burgeoned in India, resulting in an extremely large number of multidrug-resistant TB cases, second only to the number of cases noted in Latvia (2). Since 1993, in response to this epidemic, the government of India has implemented the Revised National Tuberculosis Control Program, which is based on directly observed treatment (short course) principles (1).

Mumbai (formerly Bombay), India, is a densely populated metropolis with a population of approximately 12 million, 4.8 million (40%) of whom reside in overcrowded slums. Since 1990, a resurgence of TB has occurred, characterized by a 70% to 140% increase in the rate of TB-related deaths among adults aged 25–44 years (3). A vital factor contributing to this phenomenon is HIV infection. A recent review of autopsy reports from Mumbai showed that 85 (59%) of 143 adult patients with AIDS were diagnosed with pulmonary TB (4), indicating that the disease is the most common opportunistic infection for persons with AIDS. Commensurate with the increase in TB cases is a surge in the prevalence of multidrug-resistant TB in adult patients. Two reference mycobacterial laboratories in private hospitals in Mumbai have reported a high prevalence of multidrug-resistant TB strains; 56 (11%) of 521 cases in 1991–1995, and 58 (58%) of 100 cases in 1994–1995 (5,6).

The crisis of multidrug-resistant TB in adults in Mumbai has been well documented (7). However, little attention has been directed at children also affected by the resurgent TB epidemic. We think that TB is developing in more children in Mumbai today than a decade earlier. Moreover, close proximity to adult patients with multidrug-resistant TB makes children prone to developing primary multidrug-resistant TB, a vulnerability documented in a South African study (8). Similarly, disseminated TB is occurring in large numbers of children living in overcrowded slums in Mumbai with a consequent high death rate (9); we attribute many of these deaths in children to primary multidrug-resistant TB. However, this conclusion is difficult to document, as most affected children are sputum-negative for acid-fast bacilli. Contact tracing to detect the adult source of infection is routinely undertaken; often we can trace the source of infection. Because the facilities for culture and susceptibility testing are not available at affordable rates, proving that the adult contact has multidrug-resistant TB is not feasible in most cases.

 The AIDS epidemic in adults in Mumbai has adversely affected the epidemic within the population of children with TB. HIV infection in young adults has resulted in a large number of HIV-infected infants, the result of a lack of any large-scale program aimed at preventing vertical transmission. To combat the growing problem with HIV-infected infants, India’s National AIDS Control Organization is performing feasibility studies for implementing interventions to prevent mother-to-child transmission of HIV infection. Clinical trials with nevirapine are currently being conducted at five major public hospitals in Mumbai.

Multidrug-resistant TB frequently develops in adult AIDS patients (7). Accordingly, many pediatric AIDS patients in Mumbai are also developing primary multidrug-resistant TB. Since most families cannot afford antiretroviral therapy commonly prescribed for multidrug-resistant TB, HIV-infected children in whom TB is diagnosed are prescribed a four-drug TB treatment (consisting of isoniazid, rifampicin, pyrazinamide, and ethambutol) and co-trimoxazole for *Pneumocystis carinii* pneumonia prophylaxis. Although deaths in these children are being attributed to AIDS, we think that many of these deaths are related to multidrug-resistant TB.

 To combat the TB epidemic, the Revised National Tuberculosis Control Program directly observed treatment strategy has been implemented as part of a public health program. However, most patients receive treatment from private physicians and thus remain outside the purview of the strategy. Private physicians seldom refer their patients to centers offering directly observed treatments because of potential for loss of income (3). In 1991, Uplekar and Shepard (10) reported that 100 private physicians in the Dharavi slums in Mumbai prescribed 80 different anti-TB regimens; most were both inappropriate and expensive. Since private physicians have not yet been involved in the government-run Revised National Tuberculosis Control Program, the situation today remains the same.

Although children are included in the national control program, they do not receive the benefit of directly observed treatment strategy. The Revised National Tuberculosis Control Program does not provide drugs in syrup form or permit breaking of tablets, making the administration of accurate pediatric doses impossible. Most children with TB are also sputum-smear negative for acid-fast bacilli. Doctors must rely on clinical acumen when deciding whether or not to start TB treatment. This lack of a method for definitive diagnosis of TB in children makes treatment centers reluctant to enlist pediatric cases; as a result, these children attend general pediatric outpatient clinics every 28 days to obtain their TB medication. Directly observed treatment strategy is not followed in the general outpatient clinics. Hence, compliance with treatment depends on the motivation and perseverance of the parents. Frequently, one or more of the drugs is out of stock, and parents must use their own small resources to purchase the necessary medication. To avoid long waits in the crowded general pediatric outpatient clinics, some parents intermittently purchase the anti-TB drugs from local chemists, who supply the drugs without a current prescription. This practice leads to frequent defaulting and inadequate treatment.

Since children in general have paucibacillary TB, secondary multidrug-resistant TB is considered less likely to develop in them, even when the treatment is inadequate. However, Karande et al. (11) describe a 12-year-old boy in Mumbai with secondary multidrug-resistant TB. He had received multiple courses of inadequate treatment with various anti-TB treatment regimens for 9 years. The TB gradually progressed in severity and was disseminated with the bacterial load increasing sufficiently for multidrug-resistant TB to develop. We suggest that this case is not unusual and that many children in Mumbai are dying of multidrug-resistant TB because directly observed treatment strategy regimens are unavailable.

The multidrug-resistant TB crisis on Mumbai’s children warrants immediate attention and action. We suggest that the directly observed treatment strategy should be made child-friendly with anti-TB drugs made available in suitable pediatric formulations. Private physicians require education and involvement in the treatment strategy. BACTEC (BD Diagnostic Systems, Sparks, MD) culture and susceptibility testing to detect multidrug-resistant TB should be made available at affordable rates. Transmission of HIV to newborns would be reduced by universally implementing a prevention program for mother-to-child transmission at subsidized rates. Immediate action on these suggestions will lower the incidence of both TB and multidrug-resistant TB and reduce the number of deaths from these diseases.
